# Laughter

**Published:** 1885-03

**Authors:** 


VOL. 32. MARCH 1885. NO. 3.

### LAUGHTER.

The exciting emotions which are pleasurable, such as joy and hope, are of a kind that seldom tend to dangerous excess, and may be regarded as exercising generally an eminently healthful influence upon the body. Hilarity is a great refresher and strengthener of life. Laughter is a wholesome exercise, which, beginning at the lungs, diaphragm, and connected muscles, is continued to the whole body, shaking the sides, and causing that jelly-like vibration of the frame of which we are so agreeably conscious when under its influence. The heart beats more briskly, but with a safe regularity of action, and sends the blood to the smallest and most distant vessel. The face glows with warmth and color, the eye brightens, the temperature of the whole body is moderately raised. With the universal pleasurable sensation there comes a disposition of every organ to healthy action. When hilarity and its ordinary expression of laughter becomes habitual, the insensible perspiration of the skin is increased, the breathing quickened, the lungs and chest expanded, the appetite and digestion strengthened, and nutrition consequently increased The old proverb "Laugh and grow fat" states a scientific truth. The influence of laughter upon the body is recognized by Shakspeare in his description of the "Spare Cassius—seldom he smiles." "To be free-minded and cheerfully disposed at hours of meal and sleep and exercise, is one of the best precepts of long lasting." Such is the testimony of Lord Bacon to the favorable influence of the pleasurable emotion upon the body. The depressing emotions, such as fear, anxiety and grief, are always fatal to health, and frequent causes of death. There is an Eastern apologue which describes a stranger on the road meeting the Plague coming out of Bagdad. "You have been committing great havoc there," said the traveller pointing to the city. "Not so great!" replied the Plague. "I only killed one-third of those who died; the other two-thirds killed themselves with fright."

